# Hot Spots in a Network of Functional Sites

**DOI:** 10.1371/journal.pone.0074320

**Published:** 2013-09-02

**Authors:** Pemra Ozbek, Seren Soner, Turkan Haliloglu

**Affiliations:** 1 Department of Bioengineering, Marmara University, Goztepe, Istanbul, Turkey; 2 Department of Chemical Engineering and Polymer Research Center, Bogazici University, Bebek, Turkey; Universite de Sherbrooke, Canada

## Abstract

It is of significant interest to understand how proteins interact, which holds the key phenomenon in biological functions. Using dynamic fluctuations in high frequency modes, we show that the Gaussian Network Model (GNM) predicts hot spot residues with success rates ranging between S 8–58%, C 84–95%, P 5–19% and A 81–92% on unbound structures and S 8–51%, C 97–99%, P 14–50%, A 94–97% on complex structures for sensitivity, specificity, precision and accuracy, respectively. High specificity and accuracy rates with a single property on unbound protein structures suggest that hot spots are predefined in the dynamics of unbound structures and forming the binding core of interfaces, whereas the prediction of other functional residues with similar dynamic behavior explains the lower precision values. The latter is demonstrated with the case studies; ubiquitin, hen egg-white lysozyme and M2 proton channel. The dynamic fluctuations suggest a pseudo network of residues with high frequency fluctuations, which could be plausible for the mechanism of biological interactions and allosteric regulation.

## Introduction

Proteins have crucial roles in all kinds of biological functions such as gene expression control, cellular communication and immunological response. Biological functions are regulated via protein interactions. Correct detection and understanding of protein-protein/ligand interactions are of importance first to relieve how proteins act and communicate for their function and then enhance the protein and drug design. Currently, there is neither a definite list of rules nor a general pattern describing the mechanism. More than a local phenomenon, the concept of nonlocal, long-range allosteric interactions via signaling appears as a fundamental idea of engineering proteins with desired properties [1]. Early definitions of allostery can be based on the conformational change with the binding of ligands [2], [3] and displacement of the equilibrium between conformational states [4]. The newly emerging definition emphasizes the importance of dynamics in allosteric regulation [5] with the identification of residues responsible for the dynamics and combine this with the evolutionary information [6], [7], [8], [9], [10], [11].

The prediction of complex structures is a challenging task. X-ray crystallography and nuclear magnetic resonance (NMR) spectroscopy are the experimental methods commonly used to have a detailed structural knowledge. With computational methods, the main sight is concentrated on the interface or binding site prediction. It has been found that generally only a few of interacting residues contribute at the most to the binding energy [12], [13], [14]. Experimentally, these residues can be identified by a significant reduction in the binding energy upon mutation. The residues that contribute more than 2 kcal/mol to the binding energy are conventionally defined as hot spot residues [14]. Mostly being located around the center [15] or the clefts [16] of interfaces, hot spots stabilize the complex structure [17].

Sequence conservation shown to correlate with the alanine scanning hot spots [18], [19] is a widely used property in predictions [15], [20], [21]. The propensity preferences of hot spots displayed that the most frequently observed hot spot residues are tryptophan, arginine and tyrosine, while leucine, serine, threonine and valine are the less frequent [12], [18], [22]. It was also shown that aspargine and aspartic acid are more common than glutamine and glutamic acid [12], . Hydrophobicity, solvation energy, solvent accessible surface area (SASA) and residue composition are the properties used for a simple way of differentiating interacting and noninteracting residues [23]. Protein interactions sites have also been considered as the sites of concave shapes or pockets on the surface [24], [25], [26], [27], [28], [29], [30]. Yet, there is no single property distinguishing the interacting sites from the rest of the structure.

Experimental data regarding the binding energies for a limited number of complexes is available mainly by Alanine Scanning Database (ASEdb) [12] and Binding Interface Database (BID) [31]. To complement experimental studies, computational methods are constantly being developed; using energy contribution [32], [33], [34], sequence [18], [19], [35], [36] and structure [16], [37], [38], [39], [40], [41] based information sources, mostly with learning tools [20], [36], [42], [43], [44] and simulation methods [45], [46]. Many servers are available, such as ISIS [47], FOLDEF [32], ROBETTA [33], K-FADE/K-CON/ROBETTA [42], MAPPIS [48], HotPoint [49], HotSprint [36], and pyDockNIP [50]. All are based on bound complex structures, except ISIS and pyDockNIP. ISIS is a sequence based method and has an advantage of applicability when the structure is not available as well as when the binding partner is not known. pyDockNIP is an energy based docking simulation technique. Table S1 in File S1 summarizes the data on the available servers and databases. A detailed review on the available servers is available in recent studies [17], [51].

Binding regions provide gates on the surface through which the communication is possible between biologically interacting partners. Interactions between the gates and other functional sites should be essential for both intra- and intermolecular biological signaling. The protein’s evolutionary properties suggested that active sites are related to many surface sites [52]; the binding activity at one site may affect the activity of another distinct site [10]. It was also shown that it is possible to create multi-domain allosteric systems with desired properties [1]. The understanding of allosteric control achieved through hot spots on the surface and other functional sites is of significant interest in protein mediated signaling [52].

The fluctuations in the high frequency (fast) modes by the Gaussian Network (GNM) [53], [54] signify folding core as well as binding core residues [55], [56], [57], [58], [59]. The slow modes describe the global motion and relieve the residues responsible for the collective functional dynamics; the fast modes describe localized fluctuations and the high frequency fluctuating sites are known for their resistance to conformational changes delineated by high degrees of conservation. The interaction pathways [60], [61] between functional sites could be followed by the fluctuations in the high frequency modes and these regions respond strongly to energy fluctuations [58]. Some functional residues might be active in both local and global dynamics, i.e. closely spaced to the positions of hinge sites as well as high frequency fluctuations. Here, we suggest that binding hot spots reside in a pseudo network of functional residues that underlies the dynamics and function. To this, we show that the residues fluctuating in the high frequency modes highly overlap the experimentally known hot spot residues on a dataset of unbound protein structures as well as other functional residues. Binding sites in an intrinsic network of functionally important residues may provide a dynamic infrastructure to be disclosed upon activation. For this, case studies were presented to demonstrate the correlation of known functional residues with the residue network suggested by the high frequency modes.

Relative solvent accessibility and evolutionary conservation as properties of hot spots were also revisited and analyzed with respect to the residue fluctuations in the high frequency modes.

## Materials and Methods

### Dataset

Up-to-date only a limited number of interfaces have been investigated in detail for hot spots residues. The dataset in this study is a collection of four datasets previously published [31], [32], [33], [62]: ASEdb, the Alanine Scanning Energetics Database [62]; the dataset by Kortemme et al. [33] of single mutations compiled from the databases ProTherm [63], ASEdb [62] and some additional reports; the dataset by Guerois [32] of experimentally studied mutations and mutants from ProTherm; and BID, The Binding Interface Database [31]. Besides BID, the other three datasets provide the change in the residue binding energy (**ΔΔ**G) values. BID categorizes the effect of mutations as strong, intermediate, weak or insignificant. The residues having strong interaction strengths and those having binding free energies >2 kcal/mol are considered as hot spots in the present analysis. To be consistent within the dataset, only experimental alanine mutations are included. PISCES sequence culling server [64] is used to avoid redundancy by removing proteins with sequence identity more than 25%.

The final dataset is composed of 33 unbound protein structures having a total of 4470 residues from which 173 are detected as hot spot residues (Table S2 in File S2). 3.9% of the total number of residues in a protein chain on the average is reported as hot-spot residues. Number of hot spots varies from 1 to 8 residues with a few exceptional cases of more than ten hot spots. An additional dataset is composed of the complex structures of unbound structures (Table S3 in File S1). Both contain only protein-protein interaction hot spots.

### The Gaussian Network Model (GNM)

GNM [53], [54] describes the protein structure as a simple elastic network where the alpha carbons within a cut-off radius (r_cut_) are assumed to be connected by harmonic springs.

The equilibrium fluctuations, **ΔR**_i_ and **ΔR**_j_, of residues i and j are given as 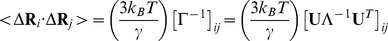
(1)Where **Γ** is the Kirchhoff connectivity matrix; **U** is the orthogonal matrix of eigenvectors (**u**_i_) and **Λ** is the diagonal matrix of eigenvalues (λ_i_); k_B_ is the Boltzmann constant and T is the absolute temperature.

The mean square distance fluctuations, <**ΔR** ^2^_ij_>, of residues i and j are given as
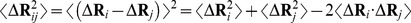
(2)Where the mobility of residues i and j and the correlation between their fluctuations are incorporated. Further details of the method are in File S1, Table S4 in File S1 and Figure S1.

### Analysis

The mean square distance fluctuations of residue i and j, <**Δ**R^2^_ij_>, in the fast modes of motion are calculated using a cutoff radius of 6.5 Å. The residues with high <**ΔR** ^2^_ij_> are considered as functionally probable. A case study is provided in File S1. The individual modes as well as the average of a number of fast modes are considered; the fastest, the second fastest and the third fastest, and the average three and five fastest modes. The performance assessments are based on the following definitions:
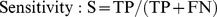
(3)

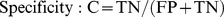
(4)

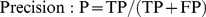
(5)

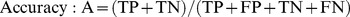
(6)where TP, TN, FP and FN stand for the numbers of true positives, true negatives, false positives and false negatives, respectively. Here, the conditional positive stands for a residue being a hot spot. Sensitivity, accuracy, specificity and selectivity have been calculated for all residues of all proteins in the dataset. The near neighbors are also considered due to the low-resolution nature of the model. The number of fast modes up to five is taken here. There is no a definite rule to *a priori* decides for the number of fast modes to be used, as this may depend on the structural and functional features. Yet, five to ten fast modes in general could be considered to capture the critical residues of fast fluctuations.

The GNM predictions are compared with the experimental binding hot spots. Additionally, the effect of relative solvent accessibility and sequence conservation effects are investigated. Relative solvent accessibility results are retrieved from Naccess [65] and the conservation data is obtained from Consurf [66]. Conservation scores varying from 1 to 9 indicates the level of evolution from a highly variable position to a position that is highly conserved.

## Results

### High Frequency Fluctuations and Hot Spots

The analyses on 33 unbound protein structures, based on the exact outcome of the fastest mode, lead to the performance values of S 14%, C 89%, P 5% and A 86% for sensitivity, specificity, precision and accuracy, respectively. Including more number of fast modes improves the sensitivity performance on the cost of specificity and accuracy. When the neighboring two residues are taken into account, the performance values for the fastest mode are: S 41%, C 90%, P 14% and A 88%, respectively. Table 1 summarizes the performance values for all modes. It should also be noted that the suggested residues are 11.3%, 12.5%, 12.6%, 14.6%, 16.9% of the overall protein structure on the average for the fastest, the second fastest and the third fastest, and the average three and five fastest modes, respectively (Table 1).

**Table 1:** The GNM performance values of the unbound dataset.

NO GNM					RSA				CONSERVATION				RSA & CONSERVATION			
					76	47	5	48	77	41	5	42	62	62	6	62
*GNM* *modes*	EXACT				EXACT& RSA				EXACT &CONSERVATION				EXACT & RSA &CONSERVATION			
	*S*	*C*	*P*	*A*	*S*	*C*	*P*	*A*	*S*	*C*	*P*	*A*	*S*	*C*	*P*	*A*
***1***	14	89	5	86	12	93	6	90	11	92	5	89	9	94	6	91
***2***	16	88	5	85	10	92	5	89	12	92	5	89	8	94	5	91
***3***	24	88	7	85	20	92	9	89	20	92	9	89	17	94	10	91
***1–3***	25	86	7	83	21	90	8	87	18	90	7	88	17	92	8	90
***1–5***	29	84	7	81	27	88	8	86	23	88	7	86	22	91	9	88
**GNM** **modes**	**NEIGHBOR 1**				**NEIGHBOR 1 &** **RSA**				**NEIGHBOR 1 &** **CONSERVATION**				**NEIGHBOR 1 & RSA &** **CONSERVATION**			
	***S***	***C***	***P***	***A***	***S***	***C***	***P***	***A***	***S***	***C***	***P***	***A***	***S***	***C***	***P***	***A***
***1***	32	90	11	87	26	93	13	91	24	93	11	90	20	95	13	92
***2***	34	88	10	86	25	93	12	90	26	92	12	90	20	95	13	92
***3***	40	89	12	87	31	93	14	90	32	92	14	90	25	94	15	92
***1–3***	39	86	10	85	32	90	12	88	30	91	12	89	25	93	12	90
***1–5***	47	84	11	83	39	89	12	87	36	89	12	87	31	91	12	89
**GNM modes**	**NEIGHBOR 2**				**NEIGHBOR 2 & RSA**				**NEIGHBOR 2 & CONSERVATION**				**NEIGHBOR 2 & RSA & CONSERVATION**			
	***S***	***C***	***P***	***A***	***S***	***C***	***P***	***A***	***S***	***C***	***P***	***A***	***S***	***C***	***P***	***A***
***1***	41	90	14	88	34	94	17	91	31	93	15	91	27	95	18	92
***2***	43	89	13	87	35	93	16	91	34	93	16	90	28	95	18	92
***3***	49	89	15	87	37	93	17	91	39	93	18	91	31	95	19	92
***1–3***	50	87	13	85	40	91	15	89	38	91	15	89	31	93	15	91
***1–5***	58	85	13	84	47	89	15	87	46	89	15	88	38	91	15	89

Labels S, C, P and A refer to sensitivity, specificity, precision and accuracy, respectively. GNM modes 1–3 and 1–5 refer to the average three and five fastest modes, respectively. The reported values are percentages.

Low sensitivity observed can be related to not being able to determine all of the hot spot residues given. Nevertheless, taking the neighboring residues into account improves sensitivity, as the high frequency fluctuating residues are mostly in clusters. Low precision observed is due to suggesting more residues as hot spot residues than the actual. When the suggested residues that do not match with the actually reported hot spot residues are investigated, most of these residues have been reported to have still relatively high binding free energies (**ΔΔ**G ≥1.5) (Figure S2) or alternatively have other functional importance. Further, the Z-score analysis displays that the high frequency fluctuating residues tend to be closer to the experimental hot spots than the rest of the residues; the distributions are shifted toward shorter distances with negative Z-scores. 65% of the predicted residues for the fastest GNM mode with two neighboring residues have a Z-score of less than−1. The details of the statistical analysis are given in File S1 and in Figures S3 and S4.

For the enhancement of predictions, relative solvent accessibility and evolutionary conservation analysis are revisited for their possible contribution to the prediction of hot spot residues by the fast dynamics.

#### Relative solvent accessibility

The relative solvent accessibility (RSA) values of hot spot residues are analyzed for 33 unbound proteins in the dataset. The free energy change values with alanine mutations [31], [32], [33], [62] versus their RSA values by Naccess [65] are plotted in Figure S5.

From a total of 509 mutation data, 28 of the cases have a RSA value equal to 0 (5.5%) with 22 of them are specified as hot spots based on the definition of binding energy change (78.5%); 282 of the cases have a RSA value equal or less than 40 (55.4%) with 137 of them are specified as hot spots (48.5%). This confirms the previous findings that there is no definite correlation between solvent accessibility and residues binding energy contribution [12], [13]. As seen in Figure S5, the residues having a **ΔΔ**G value of more than 2 kcal/mol cluster in the region where RSA values are low. A RSA value threshold of 40 is expected to increase true positives in our predictions. From the other perspective, out of 509 mutation data, 179 are hot spot residues with 137 of them having RSA values equal or less than 40. 76.5% of hot spots have thus RSA values less than 40. It should be noted that for the cases where no RSA data is available, this parameter is not applicable.

After filtering the outcome of the highest frequency mode by removing the residues having a RSA value greater than 40, the results with two residues neighboring lead to the performance values of S 33%, C 93%, P 16% and A 91% for sensitivity, specificity, precision, and accuracy, respectively. On the cost of sensitivity, the rest of the performance values are slightly improved in comparison to only dynamics based predictions (Table 1). The residues of the average five fastest modes are observed to accumulate at relatively low RSA values (See Figure S6). Nevertheless, 14.4% of the residues having low RSA values overlap the regions of the high frequency fluctuations.

#### Evolutionary conservation

The evolutionary conservations of hot spot residues are also analyzed. From a total of 509 mutation data, 310 of the cases have conservation scores equal or greater than 5 with 139 of them are specified as hot spots (44.8%). It shows that the evolutionary conservation itself might be misleading in discriminating hot spot residues from the rest [67], [68]. From the other perspective, out of 509 mutation data, 179 of them are hot spot residues with 139 of them having conservation scores equal or greater than 5. 77.7% of hot spots are conserved with a score of at least 5.

When the evolutionary conservation is taken into account by considering the predicted residues having a conservation score of 5 or more, the results based on the highest frequency mode and two residues neighboring give the performance values of S 31%, C 93%, P 15% and A 91% for sensitivity, specificity, precision, and accuracy, respectively. On the cost of sensitivity, the performance values of the rest are slightly improved (Table 1). On the other hand, the residues that appear in the fast modes of motion as demonstrated for the average five fastest have high conservation scores (Figure S7) in line with the previous studies [51], [69].

#### Relative solvent accessibility versus evolutionary conservation

Out of 4470 residues, 2399 of them have a RSA value smaller thanr equal to 40 (53.6%) with 1727 of them are being conserved (72%) and 110 of them are specified as hot spots based on the definition of binding energy change (6.37%). From the evolutionary conservation point of view, out of 4470 residues in total, 2665 of the cases have conservation scores equal or greater than 5 (59.6%), 1727 of the conserved residues have a RSA values smaller than or equal to 40 (64.8%) and 110 of them are specified as hot spots based on the definition of binding energy change (6.37%). In addition to the dynamics, by taking the RSA and evolutionary conservation values into account, S 27%, C 95%, P 18% and A 92% are achieved for sensitivity, specificity, precision and accuracy, respectively, based on the fastest mode with two residues neighboring (Table 1).

When the relative solvent accessibility and conservation parameters are considered individually for all residues in the dataset, out of 4470 residues, 2399 of them have RSA value less than 40 from which 135 are hot spot residues (5.6%). Out of 4470 residues, 2665 of them have conservation score of equal or more than 5 from which 137 are hot spot residues (5.1%). This is an indication that these parameters alone are not sufficient in predicting hot spot residues.

RSA and conservation analyses were performed as a filter, thus it doesn’t lead to an increase in true positives but can decrease false positives for some of the cases. Thus, we observe a slight increase in the precision but also a slight decrease in sensitivity. As the results present, the high frequency fluctuating residues correlate with the relative solvent accessibility and RSA values and the incorporation of these properties do not significantly contribute to the prediction performance values.

#### Unbound versus bound conformations

The GNM predictions are also tested on the co-crystal (bound) conformations of the chains. The analysis is performed on nine structures in the dataset having a total of 39 hot spot residues (Table S3 in File S1). Here, the analysis was performed on the bound conformations without and with the interface information. For the former, based on the average five fastest mode with two residues neighboring performance values of S 51%, C 83%, P 9% and A 82% for sensitivity, specificity, precision and accuracy, respectively (Table S5 in File S1). Relative solvent accessibility and conservation parameters have similar effects on the performance values. The performance values are very similar to those of unbound conformations, despite the conformational changes with RMSD differences ranging from 0.41 to 2.95 Å. As was shown previously, the residues that appear in the fast modes of motion in unbound and complex conformations overlap significantly [59]. This holds a proof to that the high frequency fluctuating residues are those which resist to the conformational changes at the most and stand with a predefined dynamic property. On the other hand, for the latter, based on the average five fastest modes with two residues neighboring, the performance values are S 51%, C 96%, P 30% and A 95% for sensitivity, specificity, precision and accuracy, respectively (Table S6 in File S1). The interface information decreases the number of FPs and increases specificity, precision and accuracy. 79% of the predicted residues for the fastest GNM mode with two neighboring residues have a Z-score of less than−1. The results of the analysis are given in Figures S8 and S9.

For both unbound and bound conformations, the calculations were performed on isolated chains. When the calculations were performed on the complex structures, including both interacting chains, the residues that fluctuate in the fast modes highly overlap those of the isolated bound conformations. This shows that these sites are able to display high frequency fluctuations without the contribution of the interacting chain.

#### Comparison of the GNM method with the others

The GNM approach proposed is based on unbound conformations and does not require interacting partners. There have not been much detailed studies on unbound structures: ISIS [20] is a sequence based method and pyDockNIP [50] is an energy based docking simulation technique that yet still needs interacting partners. A direct comparison is carried between the GNM method and ISIS [20] using the same dataset (Table S2 in File S1). The performance values of ISIS are S 8%, C 90%, P 3% and A 87% for sensitivity, specificity, precision and accuracy respectively. GNM performs better at all modes and conditions with respect to sensitivity and precision. Nevertheless, the comparison might also not be quite fair, as ISIS has the advantage of not requiring structure information.

On the other hand, the GNM prediction performance values with a single property on bound conformations appear comparable to those of other available methods (Table S1 in File S1). The reported values vary between S 15–78% for sensitivity, C 71–91% for specificity, P 53–89% for precision, and A 68–78% for accuracy. It should be noted that the methods use different definitions of hot spot residues and are trained on different datasets while using different standard of performance measurements.

### Hot Spots in a Network of Plausible Functional Residues

#### Ubiquitin

Ubiquitin is a monomeric protein related to approximately 100 proteins [70]; it has a significant role in signaling events. ILE 44 located in a hydrophobic patch is a highly conserved residue and significantly contributes to the UIM binding of Vps 27 [71], [72]. Together with HIS 68, ILE 44 is considered to be the central binding hot spot [70], [73]. ILE 36 was shown to take part in the ternary complex formation of ubiquitin with E2/E3 [74] and considered to be an “alternate interaction site” [70]. TRABID, an OTU domain enzyme, is the first LYS 29 specific enzyme that was shown to cleave LYS 29 and LYS 33 linkages [75].

Recently, long range correlated motions in ubiquitin was demonstrated by the NMR spectroscopy [76]. With the hydrogen-bonded residue pairs of ILE 13-LEU 67, LYS 6-PHE 45, ILE 13-PHE 45 and THR 14-PHE 45, a path of dynamic motion was shown between ILE 13 and PHE 45 following a non-sequential correlation route of ILE 13, VAL 5, LYS 6, HIS 68, ILE 44, ILE 45 [76]. These sites coincide the binding interface for ubiquitin binding domains (UBDs); the loop between strands β1 and β2, the C-terminal end of strand β5, and the loop between strands β3 and β4 [77] linking the residues that are functionally important [78]. ILE 13 and VAL 70 were shown to play an important role in the Ubiquitin’s molecular recognition events. It was also shown that the rigidity of the structure is maintained by the packing of HIS 68 with LEU 67 and LEU 69 into the protein core [78].

**Figure 1:**
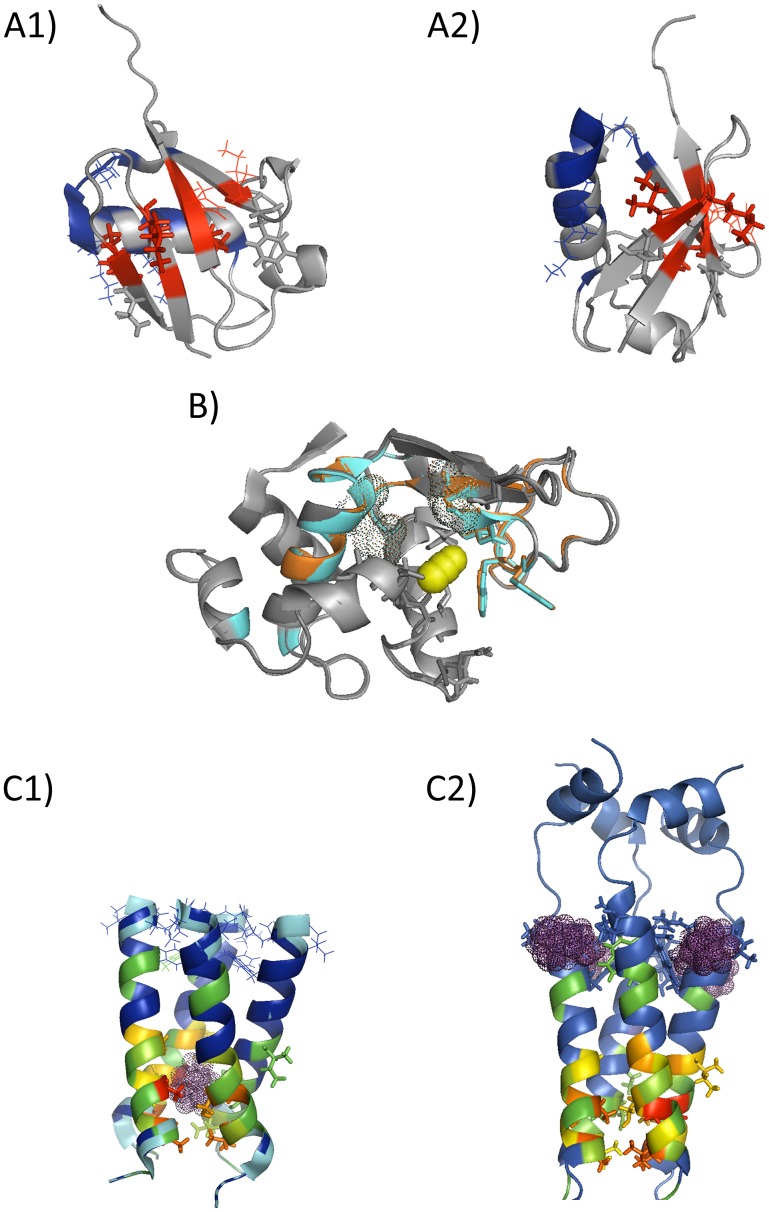
(A1 & A2) The GNM analysis performed on Ubiquitin (1 D3Z [104]). Experimentally determined hot spot residues (lines) and the long range interactions (sticks) are shown with the exact outcome of the fastest mode (blue) and the second fastest mode (red). Details are in Figure S10. (A1) and (A2) display the same figure from two perspectives. (B) The residues fluctuating in the high frequency modes by GNM for the unbound (dark grey: 2****LYM [89]) and bound (light grey: 2****LYO [83]) hen egg-white lysozyme (HEWL) structures in orange and in cyan, respectively. Experimentally determined hot spot residues (sticks), ligand (yellow sphere), and catalytic residues (dots) are also shown. See Figure S11. (C1 &C2) The GNM analysis performed on Influenza virus M2 proton channel, 3****BKD [95]: (C1) the amantadine bound structure 2****KQT [105] and (C2) the rimantadine bound 2****RLF [91]. The exact outcome of the fluctuations in the average five fastest modes above the threshold is colored based on the strength of fluctuations in the decreasing order (red to green). Blue display the residues below the threshold. Rimantadine and amantadine are shown in magenta dots with the corresponding sites in lines and in sticks, respectively. See Figure S12.

The exact outcome of the fastest, second fastest and the average three fastest GNM modes together with the known functional sites are presented in Figure S10. While the binding sites start revealing in the fastest mode, the residues that take part in the long-range interactions and in the correlation route appear in the second fastest mode. The average three fastest modes identify all known binding hot spots as well as the residues in the allosteric route. The details are shown in Figure 1 (A1&A2).

#### Hen egg-white lysozyme (HEWL)

Hen egg-white lysozyme (HEWL) has widely been studied for its hot spot residues by various experimental and computational studies [79], [80], [81], [82], [83], [84]. HEWL is a single polypeptide that can bind up to six saccharide units in subsites A, B, C, D, E and F at the active site. The most important binding sites are C, B, D, and A in the order of higher to lower affinity [79], [85], [86], [87]. Other small organic molecules were also observed to bind site C with the highest occupancy [81]. Experimentally determined hot spots [79], [83], [85], [86], [87] and key catalytic residues [83] are given in Figure S11.

Among various HEWL structures, we have applied GNM to2****LYO [83] with a single ligand (CCN, acetonitrile) bound at GLN 57, ILE 58, ASN 59, TRP 63, ALA 107 and TRP 108 [88]. The highest frequency mode manages to detect the catalytic site ASP52 and the selective key binding sites ASP 52, ASN 59, TRP 63. The latter two residues are at site C, the hot spot with the highest binding affinity (Figure S11). The exact outcome of the average three fastest modes detects the catalytic sites and the near neighbors. In the next fast modes (modes 5, 6, 9 and 10), the hot spot ALA 110 starts revealing as well. The corresponding ligand unbound structure is2 LYM [89]. The average three fastest modes gives the catalytic site and the near neighbors of the hot spot residues. In the next fast modes (4, 9 and 10), the hot spot ALA 110 appears as well (Figures 1 (B) and S11). On the other hand, LYS 33, ASN 60, ARG 62–TRP 64, ASN 66, ALA 76, HIS 78, ALA 108, TRP 109, and TRP 112 of human lysozyme (corresponding to LYS 33, ASN 59, ARG 61-TRP 63, ASN 65, LEU 75, ASN 77, ALA 107, TRP 108 and TRP 111, respectively in HEWL) were experimentally determined to take place in both binding and aggregation reactions [90]. Human and hen egg lysozymes with negligible RMSD differences (varying below 0.5 Å) have identical GNM fluctuation behavior. The region ASN 74-CYS 80 suggested by the fast modes of HEWL thus also appears to be functional.

#### Influenza virus M2 Proton Channel

The M2 protein is a proton channel; a homotetramer in the viral envelope of the influenza A virus activated at low pH. Adamantine-based antiviral drugs, amantadine and rimantadine, are commonly used to inhibit the channel activation. M2 gene has gained resistance to these drugs [91] and the commonly recognized drug resistant mutation for amantadine is SER31ASN [92]. HIS 37 is the pH sensor and TRP 41 is the gate [93], [94]. ASP 44 and ARG 45 forming a salt bridge are the integral parts of the channel gate. Lowering the pH affects HIS 37 and destabilizes the packing of the helices. This breaks the interaction of TRP 41 and ASP 44 and leads to the gate opening [91]. These residues and adjacent residues are hence the drug targets. Mostly, the polar ends of drugs are designed towards HIS 37 [95]. Drugs stabilizes the closed state [91], [95] and the drug resistant mutations aims to destabilize this conformation. The other mutations recognized for the drug resistance are: LEU26PHE, VAL27ALA, ALA30THR, GLY34GLU and LEU38PHE, which are spread all around the structure implying an allosteric mechanism therein [96].

The primary amantadine binding hot spot residues on M2 are VAL 27, ALA 30, SER 31 and GLY 34 in the middle of the pore [95]. The other hot spots are within the pore between residues ILE 33 and HIS 37. On the other hand, the rimantadine was shown to bind to: TRP 41, ASP 44 and ARG 45 on the outer surface of the channel [91]. The primary binding site is in the pore, while exterior binding occurs when the conditions are appropriate [97]. Allosteric relationship between ASP 44 and SER 31 was experimentally shown [98], where ASP 44 interacts with TRP 41.

Shown with the present analysis, the fastest mode covers all amantadine binding hot spots and the average five fastest modes also covers some of the rimantadine binding sites or its first neighbors (Figures 1 (C1&C2) and S12). Interesting to note that the fluctuations in the fast modes of motion is stronger for the amantadine binding site more than the rimantadine binding sites, in line with the relative observed functional standing of the two binding sites.

## Discussion

### Hot Spots Prediction Based on Residue Fluctuations: A Mechanistic View

High specificity and accuracy in hot spot predictions are observed based on the residue fluctuations in the highest frequency mode. Yet, the lower sensitivity values increase with the increase in the number of fast modes and also near neighbor residues considered. The relatively lower precision values are due to the similar dynamic behavior of other residues which could also be functional. On the other hand, a more extensive experimental alanine-scanning mutation data could help to draw better conclusions for the assessment of predictions. Most of the alanine scanning data is based on the experiments done on the anticipated sites rather than the whole structure. A protein might be interacting with multiple proteins and might have multiple interaction surfaces. [99]. A protein might use the same or different hot spots while binding to different partner proteins; hot spots could be partner specific [9]. The data for a protein interaction might not be complete in terms of defining all possible hot-spots involved. Neighboring residues might also contribute to the binding energy [44], so do likely allosteric sites.

The performance values obtained for hot spot residue predictions by the dynamics of unbound versus bound protein structures imply that the energetically critical binding sites are intrinsically predisposed. The hot spot residues fluctuating in the high frequency modes demonstrate their nature of being tightly packed and the centers of localization of the energy. The residues that appear in the high frequency modes, even up to the tens of fastest modes, only comprise a small number of residues in the form of clusters as well as some residues along possible interaction paths. The relationship between binding sites, protein topology and correlated paths of energy and fluctuations were recently described [58], [61], [100]. The slow modes describe the global motion and the residues that are active in these collective dynamics of overall structure and thus functional motion. Some functional residue might be active in both local and global dynamics, i.e. closely spaced to hinge positions and as well as the positions of high frequency fluctuations. This is a property with kinetic and thermodynamic significance closely linked to the structure’s topology, which is not only limited to the behavior of hot spots studied here but also to other functional sites. The high conservation scores of these residues also implies for their functional importance. The high frequency fluctuations possibly provide a mechanistic infrastructure that underlies the functional motion.

### Hot Spots in a Network of Functional Sites

Fast modes of motion reveal hot spots in a network of functionally important residues. This suggests an intrinsic dynamics for the structure where the residues with localized fluctuations play key roles. While the fastest mode appears to spell important functional sites, incorporation of the next fastest modes could still relieve other residues of functional importance. Different number of fast modes most probably up to ten may need to be considered, depending on the structure and function. This is demonstrated with the case studies presented.

For Ubiquitin, which is a signaling protein, the binding hot spot residues are predicted in the fastest mode, whereas the residues in the long range correlated motions that allosterically link the residues to the binding interface have correctly been determined in the second fastest mode. The average three fastest modes manage to detect the catalytic sites and the selective key binding sites for hen egg-white lysozyme (HEWL), while other hot spot residues have also correctly been identified in the next fast modes. In M2 protein, the first mode covers all amantadine binding hot spots, while the average five fastest modes introduces also some of the allosteric rimantadine binding sites. The fluctuations at the fast end of the dynamic spectrum are likely reminiscent of functionally important sites. Moreover, the fast modes being associated with stability [57] imply the significance of these residues in relation to stability.

The mode here refers to the eigenmodes of an unperturbed linear system and the modes are independent of each other, where no dissipation appears from one to another. In this respect, although non linearity is of importance, such as discussed for forming discrete breathers and independent rigid segment for allosteric interactions [101], [102], [103], energy fluctuations in proteins could still be discussed in a linear dynamic model [61]. In real systems, some perturbation may cause the energy transfer from one mode to another with a mode coupling. To this end, we suggest a pseudo network of interacting residues based on a number of fast modes. The couplings between fast modes and also some functional slow (cooperative) modes are likely to be effective in the mechanism of biological interactions and allosteric regulation.

## Conclusion

The dynamics in the fast modes of motion predict hot spot residues together with other functional residues. Based on a single dynamic property, a likely prediction for functional residues should infer that there is a general mechanism inbuilt within the protein’s topology and provide a mechanistic view. Localized fluctuations should provide a convenient means for those residues to participate in their functional motion and interactions, which may have some implications for biological communication and signaling.

## Supporting Information

Figure S1 **The GNM results for the fastest (a) and the average three fastest (b) modes of motion for 1 fkb.** Red dots represent the experimentally determined hot spot residues.
(TIF)

Figure S2 **The frequency of residues fluctuating in the average five fastest GNM modes versus the free energy (ΔΔG (kJ/mol)) change values with alanine mutations** **[32]**, [33], **[62**]. (The bar on 0.5 represents the values between the cases where ΔΔG is below 0.5, the bar on 1 represents cases where ΔΔG is between 0.5 and 1.)
(TIF)

Figure S3 **Z-score analysis results for the fastest mode of GNM with two neighboring residues on the unbound dataset.** (The bar on−3.25 represents cases having Z-score between−3.5 and−3, the bar on−2.75 represents cases having Z-score between−3 and−2.5.)
(TIF)

Figure S4 **Z-score values of the residues by the GNM predictions (blue shaded area) and the rest of residues (red shaded area) for the unbound dataset.**
(TIF)

Figure S5 **The free energy (ΔΔG (kJ/mol)) change values with alanine mutations** **[32]**, [33], **[62]** **versus their Relative Solvent Accessibility (RSA) values** **[65]** **.**
(TIF)

Figure S6 **The frequency of residues fluctuating in the average five fastest GNM modes versus Relative Solvent Accessibility (RSA) values** [**65**] **.** (The bar on 0 represents cases where the value of relative surface accessibility is 0, the bar on 10****represents cases where the value of relative surface accessibility is between 0 and 10.)
(TIF)

Figure S7 **The frequency of residues fluctuating in the average five fastest GNM modes versus the residue conservation scores from Consurf** [**66**] **.**
(TIF)

Figure S8 **Z-score analysis results for the fastest GNM mode with two neighboring residues on the complex dataset.** (The bar on−3.25 represents cases between−3.5 and−3, the bar on−2.75 represents cases between−3 and−2.5.)
(TIF)

Figure S9 **Z-score values of the residues by the GNM predictions (blue shaded area) and the rest of residues (red shaded area) for the bound dataset.**
(TIF)

Figure S10 **The GNM analysis performed on Ubiquitin, a monomeric protein with 76 residues (1 D3Z** [**104**] **).** Experimentally determined hot spot residues [70], [71], [72], [73], [74], residues taking part in the long range interactions [104] and in the correlation route [76], [78], and that have a role in structural rigidity [78] are shown. The GNM suggested sites of the fastest mode and the average three fastest modes are marked.
(TIF)

Figure S11 **The GNM analysis performed on hen egg-white lysozyme (HEWL), a single polypeptide of 129 amino acids with the unbound (dark grey: 2 LYM** [**89**] **) and bound (light grey: 2 LYO** [**83**] **) structures.** Experimentally determined hot spot residues [79], [85], [86], ligand binding sites and catalytic residues [83] are shown. The GNM suggested sites of the fastest mode and the average three fastest modes for2****LYM and2****LYO are marked.
(TIF)

Figure S12 **The GNM analysis performed on Influenza virus M2 proton channel, 3 BKD** [**95**] **. 3 BKD** [**95**] **is the drug unbound structure with four chains of 26 residues (22–47) each.** 2****KQT [105] is the solid state NMR structure of the amantadine bound M2 protein with four chains of 25 residues (22–46) each. 2****RLF [91] is the rimantadine bound M2 protein structure with four chains that have 38 (23–60) residues each. Amantadine [95] and rimantadine [96] binding sites, and allosteric sites [98] are shown. The GNM suggested sites of the fastest mode and the average five fastest modes are marked.
(TIF)

File S1 **Supplementary Data.**
(DOCX)
