# Supplementary figures and images for: Hot Spots in a Network of Functional Sites
Source: PLoS One. 2013 Sep 2;8(9):e74320. doi: 10.1371/journal.pone.0074320 (PMC3759471; doi:10.1371/journal.pone.0074320)

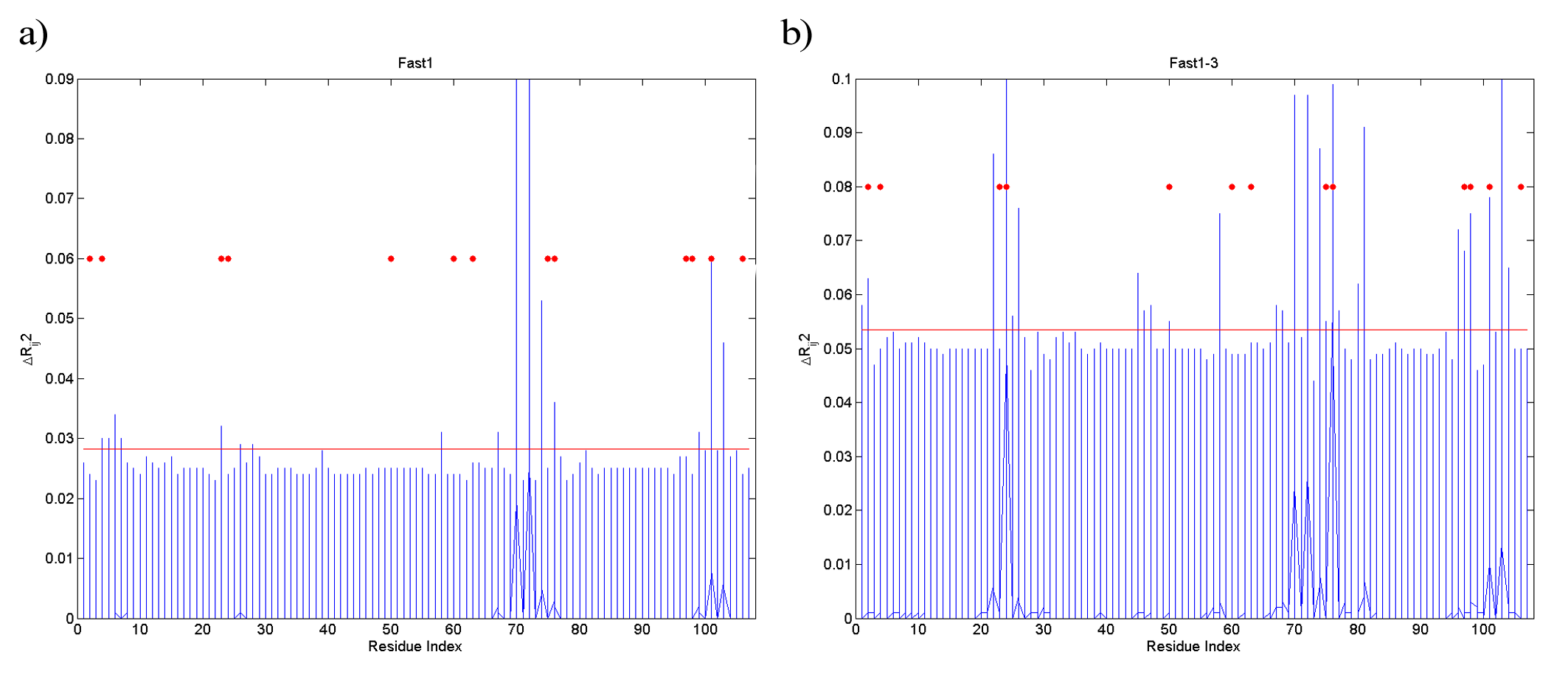

Supplement: Figure S1 — The GNM results for the fastest (a) and the average three fastest (b) modes of motion for 1 fkb. Red dots represent the experimentally determined hot spot residues. (TIF) [file pone.0074320.s001.tif]

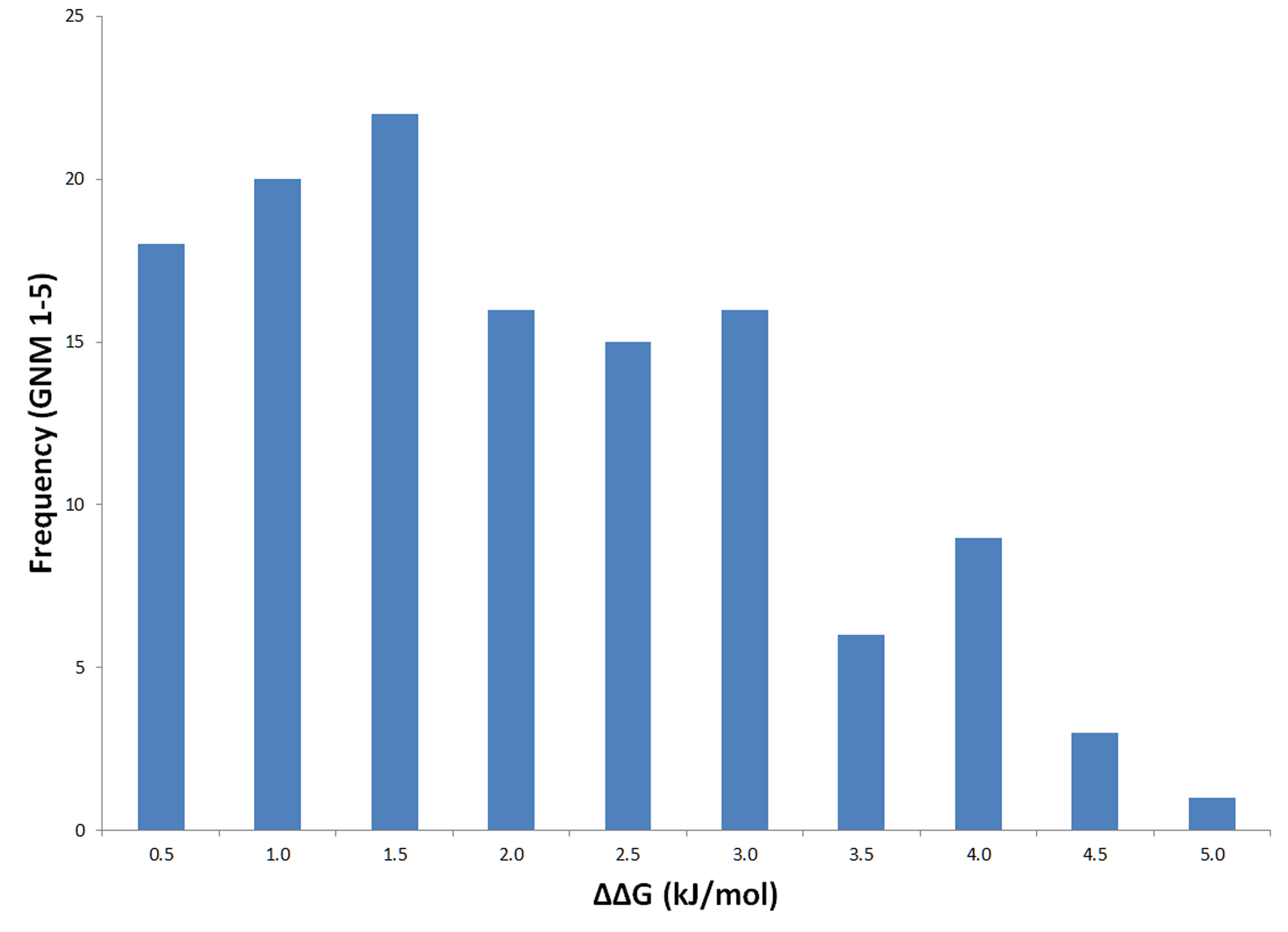

Supplement: Figure S2 — The frequency of residues fluctuating in the average five fastest GNM modes versus the free energy (ΔΔG (kJ/mol)) change values with alanine mutations [32] , [33], [62]. (The bar on 0.5 represents the values between the cases where ΔΔG is below 0.5, the bar on 1 represents cases where ΔΔG is between 0.5 and 1.) (TIF) [file pone.0074320.s002.tif]

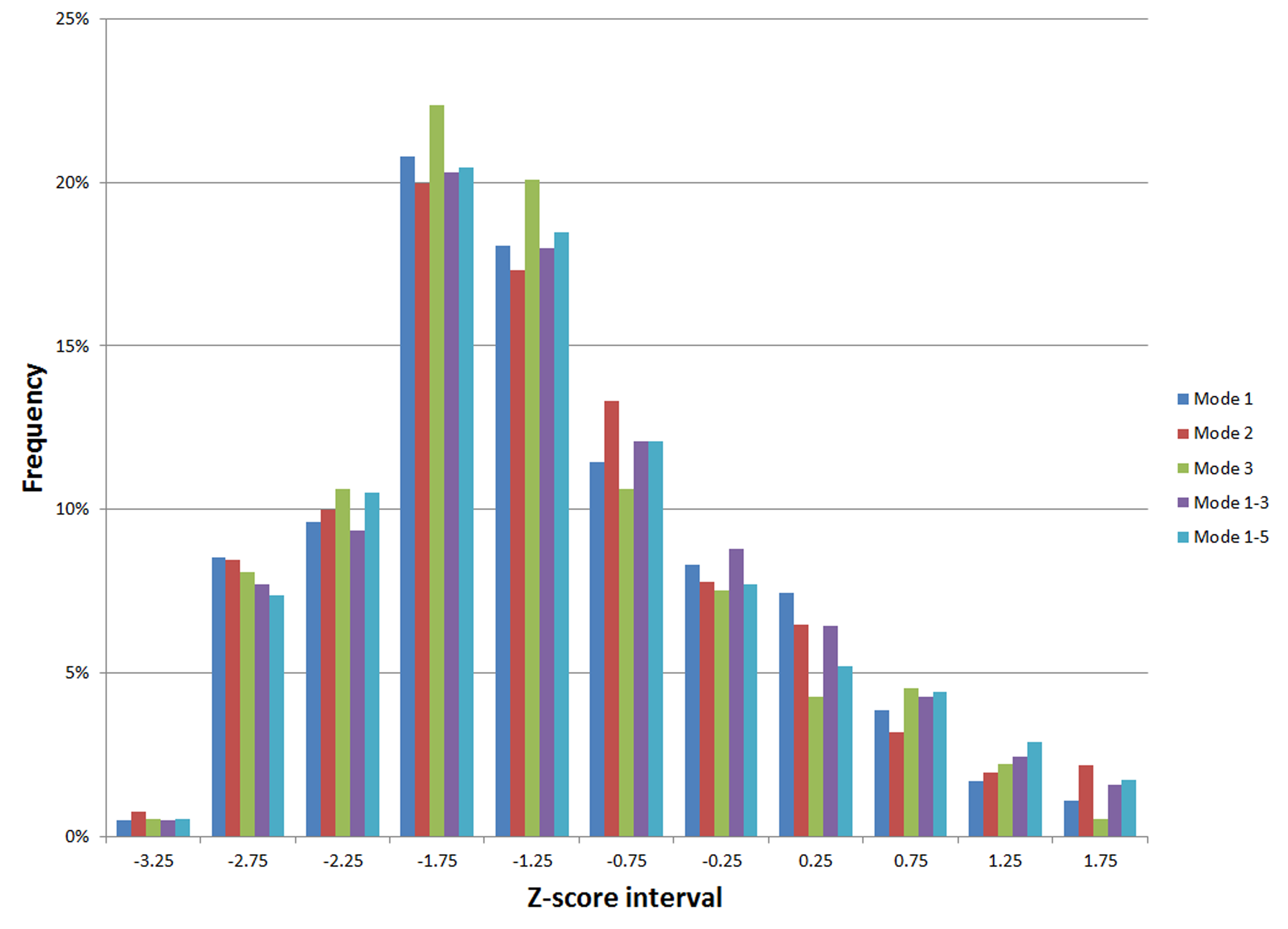

Supplement: Figure S3 — Z-score analysis results for the fastest mode of GNM with two neighboring residues on the unbound dataset. (The bar on−3.25 represents cases having Z-score between−3.5 and−3, the bar on−2.75 represents cases having Z-score between−3 and−2.5.) (TIF) [file pone.0074320.s003.tif]

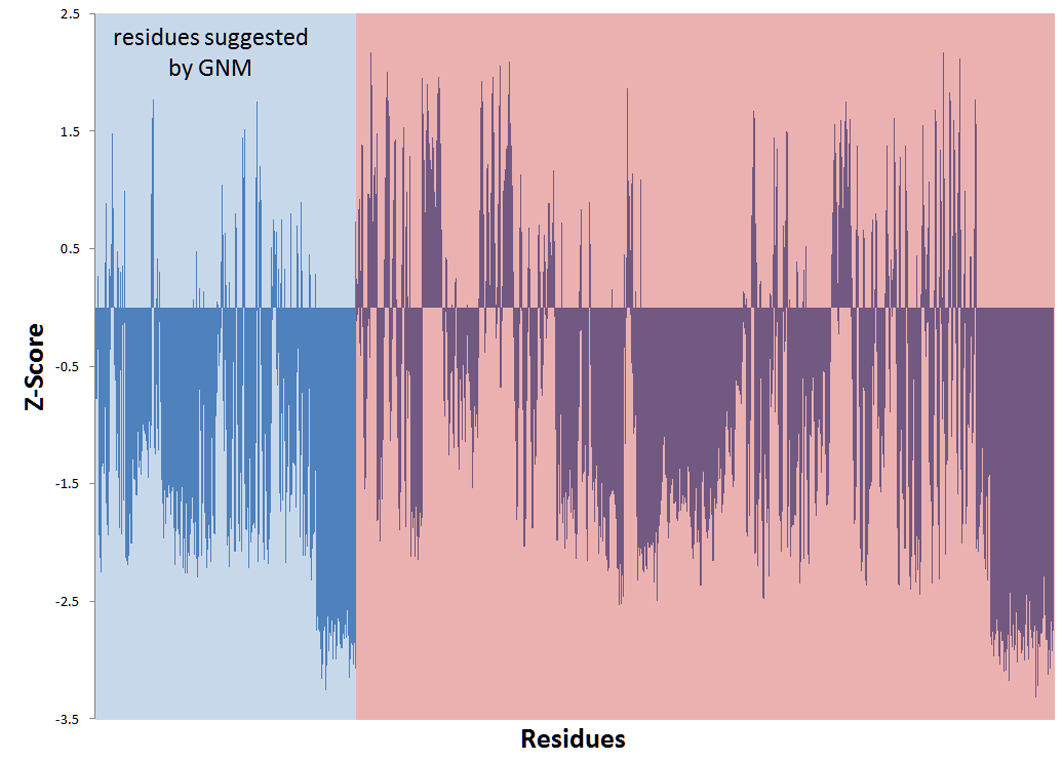

Supplement: Figure S4 — Z-score values of the residues by the GNM predictions (blue shaded area) and the rest of residues (red shaded area) for the unbound dataset. (TIF) [file pone.0074320.s004.tif]

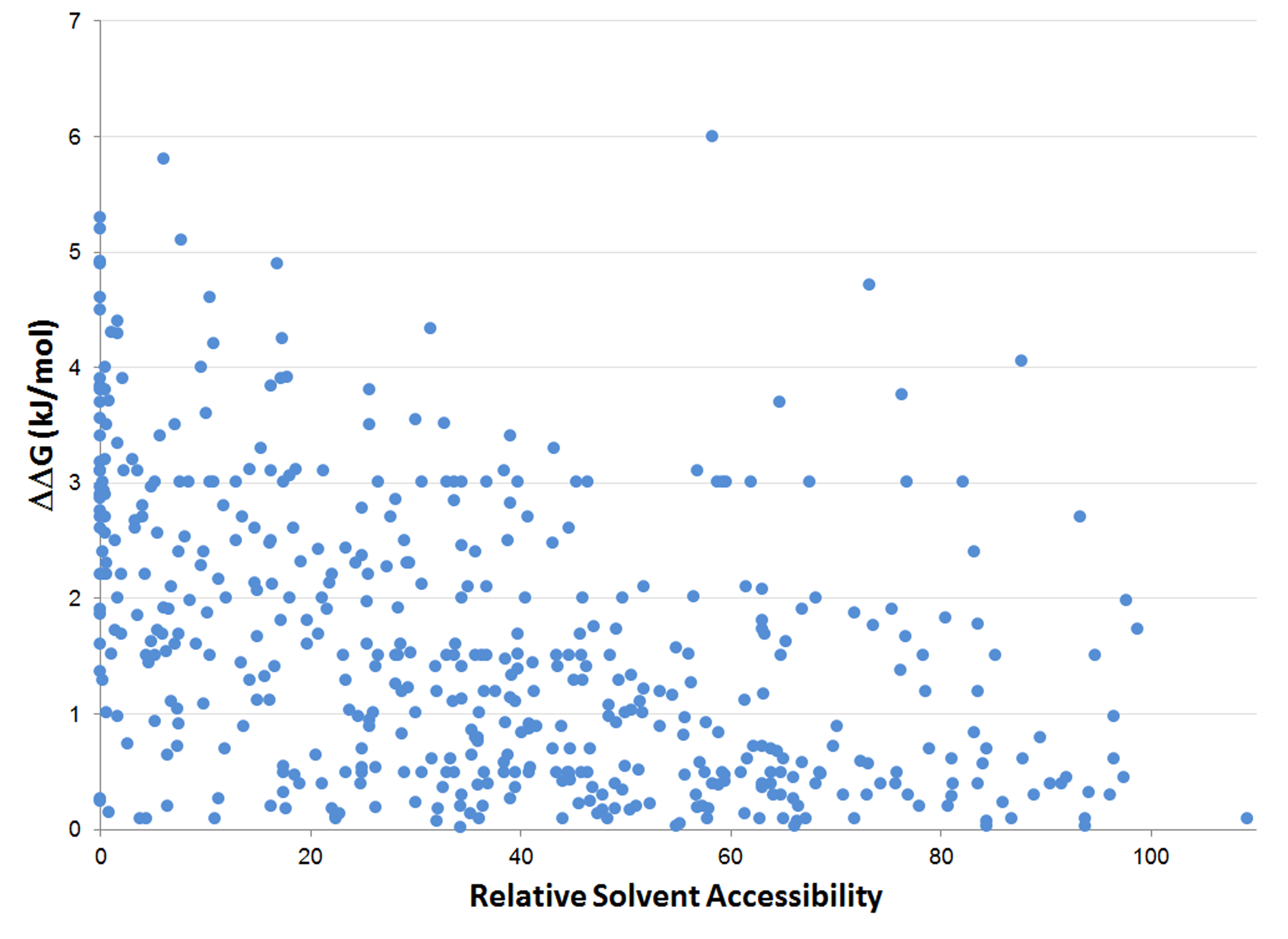

Supplement: Figure S5 — The free energy (ΔΔG (kJ/mol)) change values with alanine mutations [32] , [33], [62] versus their Relative Solvent Accessibility (RSA) values [65] . (TIF) [file pone.0074320.s005.tif]

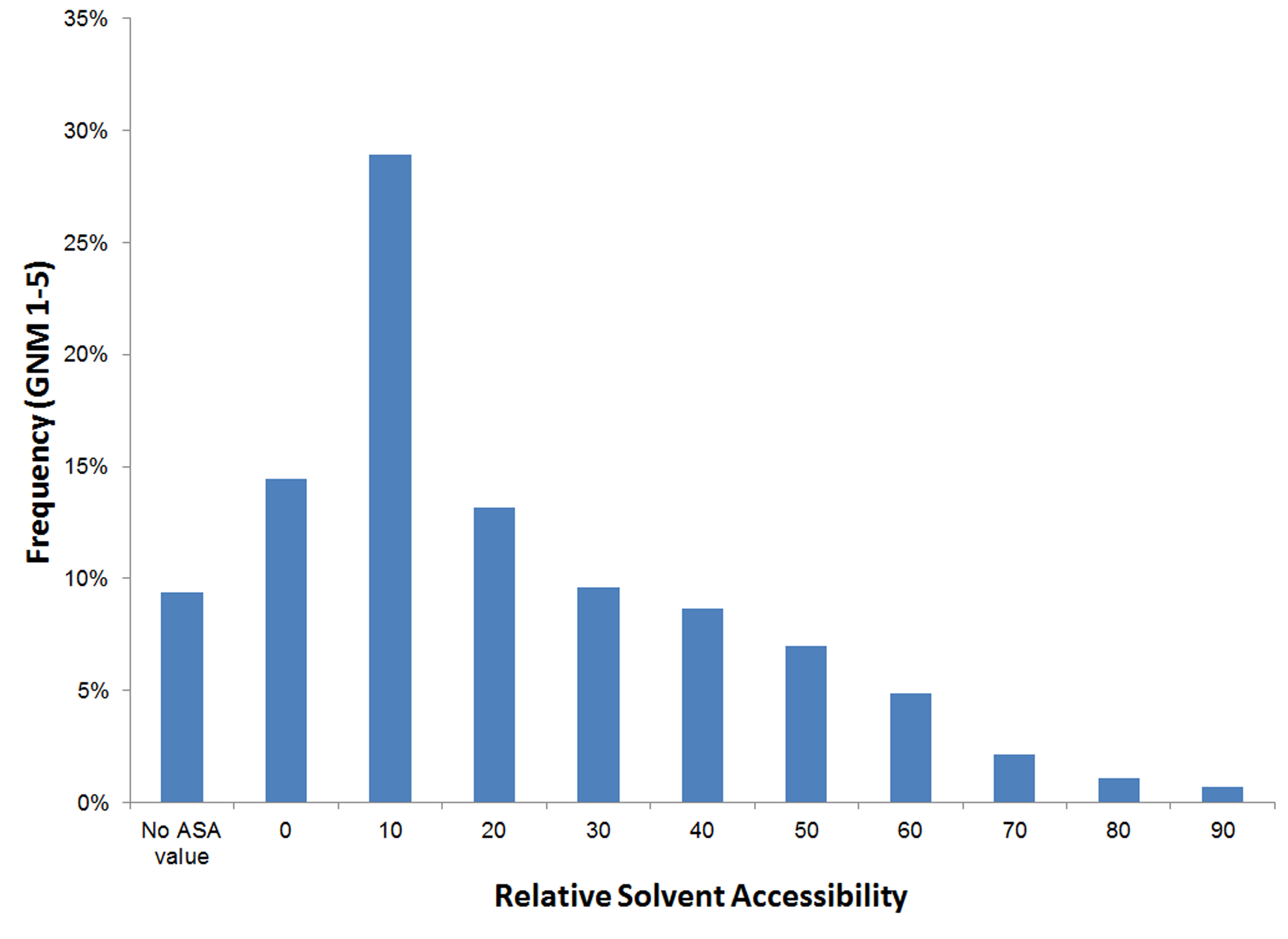

Supplement: Figure S6 — The frequency of residues fluctuating in the average five fastest GNM modes versus Relative Solvent Accessibility (RSA) values [65] . (The bar on 0 represents cases where the value of relative surface accessibility is 0, the bar on 10represents cases where the value of relative surface accessibility is between 0 and 10.) (TIF) [file pone.0074320.s006.tif]

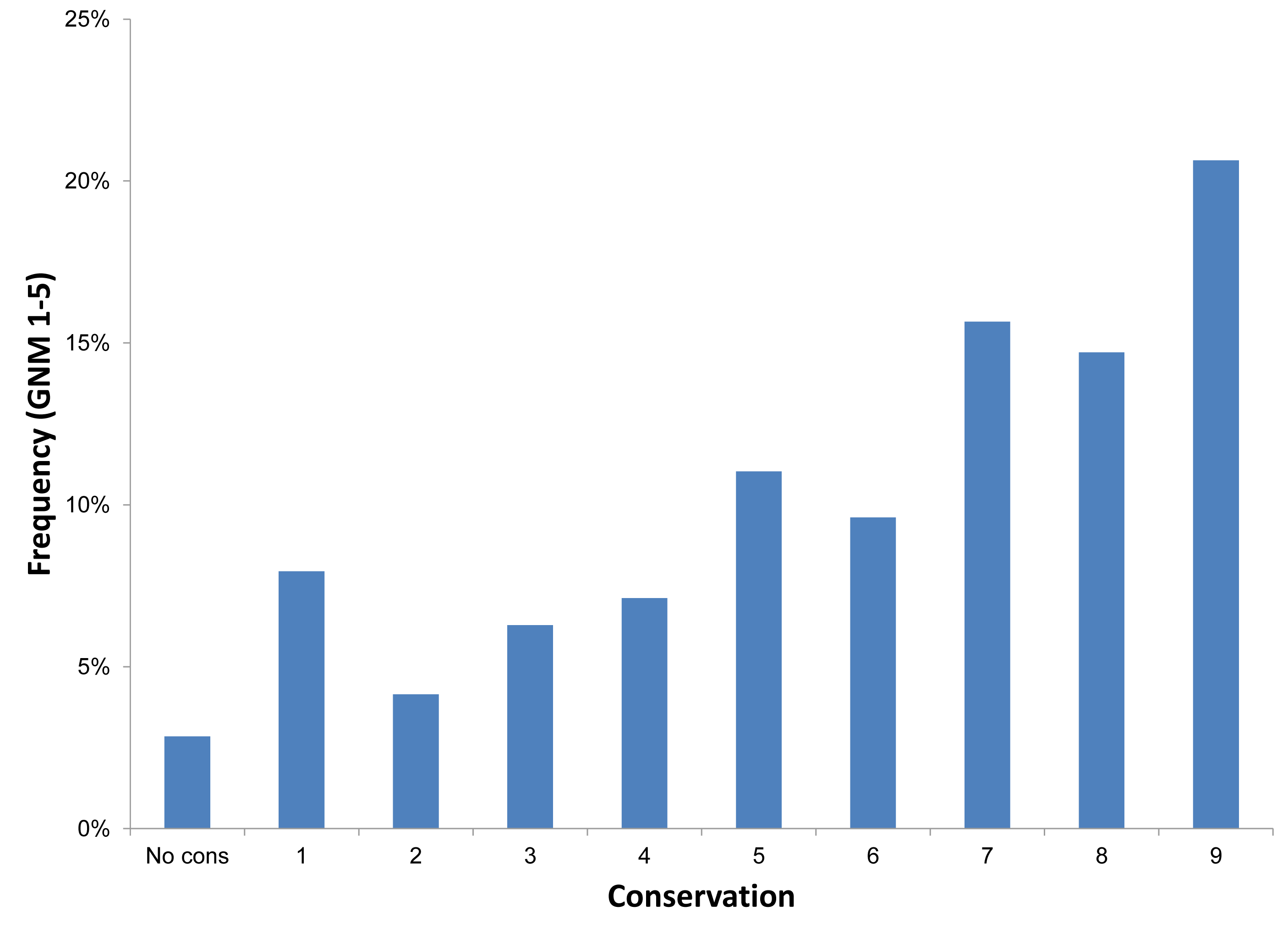

Supplement: Figure S7 — The frequency of residues fluctuating in the average five fastest GNM modes versus the residue conservation scores from Consurf [66] . (TIF) [file pone.0074320.s007.tif]

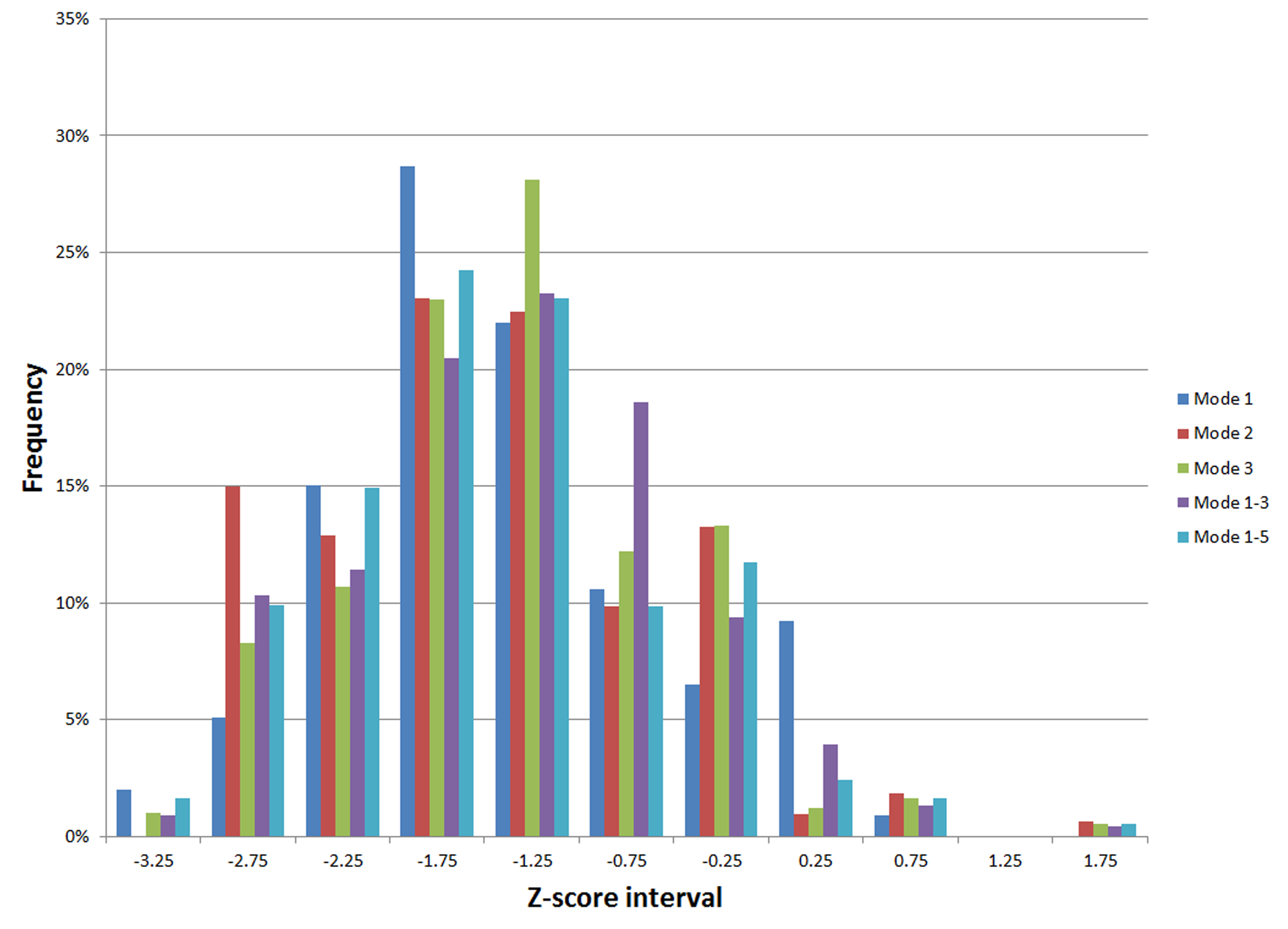

Supplement: Figure S8 — Z-score analysis results for the fastest GNM mode with two neighboring residues on the complex dataset. (The bar on−3.25 represents cases between−3.5 and−3, the bar on−2.75 represents cases between−3 and−2.5.) (TIF) [file pone.0074320.s008.tif]

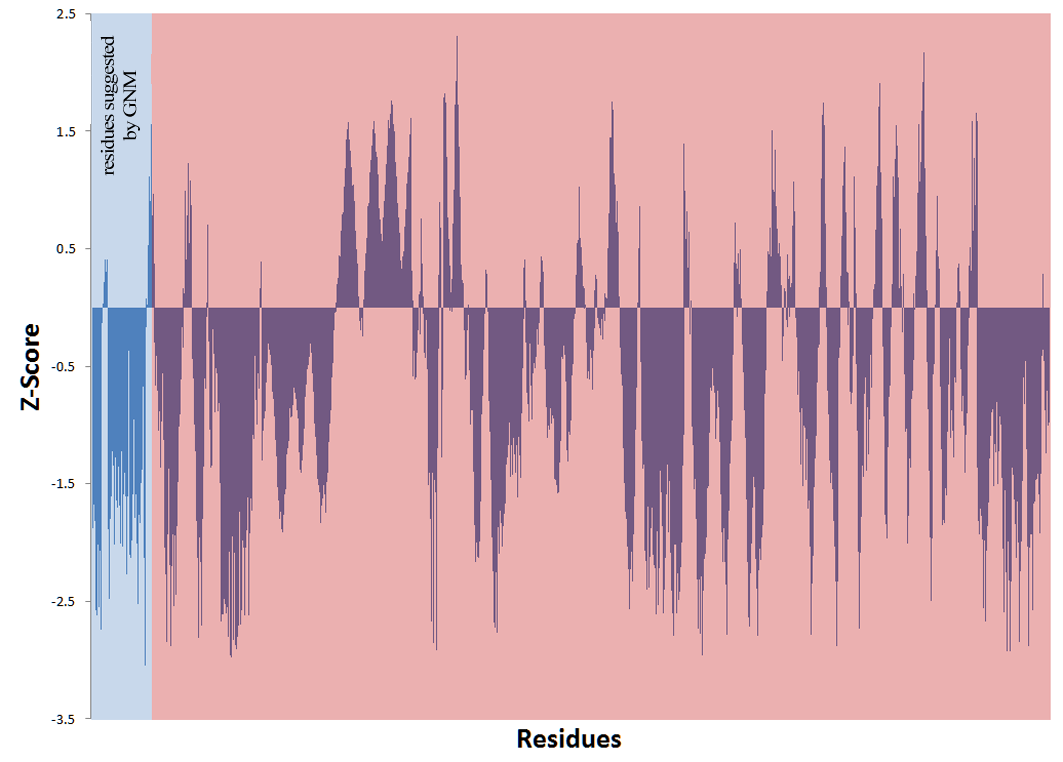

Supplement: Figure S9 — Z-score values of the residues by the GNM predictions (blue shaded area) and the rest of residues (red shaded area) for the bound dataset. (TIF) [file pone.0074320.s009.tif]

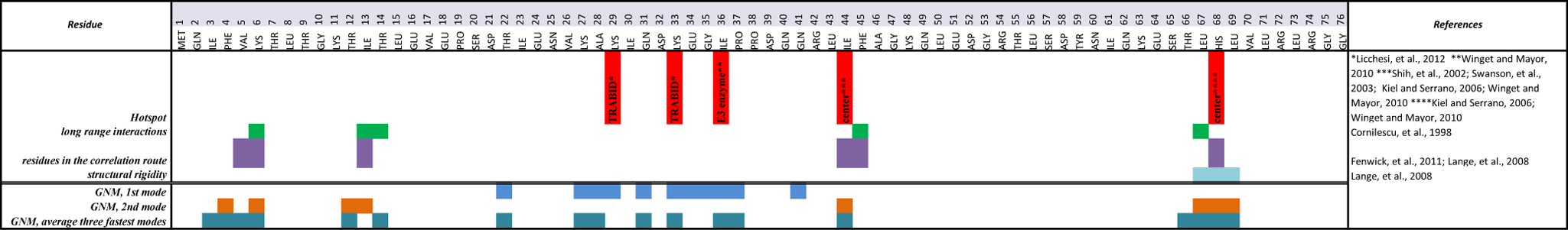

Supplement: Figure S10 — The GNM analysis performed on Ubiquitin, a monomeric protein with 76 residues (1 D3Z [104] ). Experimentally determined hot spot residues [70], [71], [72], [73], [74], residues taking part in the long range interactions [104] and in the correlation route [76], [78], and that have a role in structural rigidity [78] are shown. The GNM suggested sites of the fastest mode and the average three fastest modes are marked. (TIF) [file pone.0074320.s010.tif]

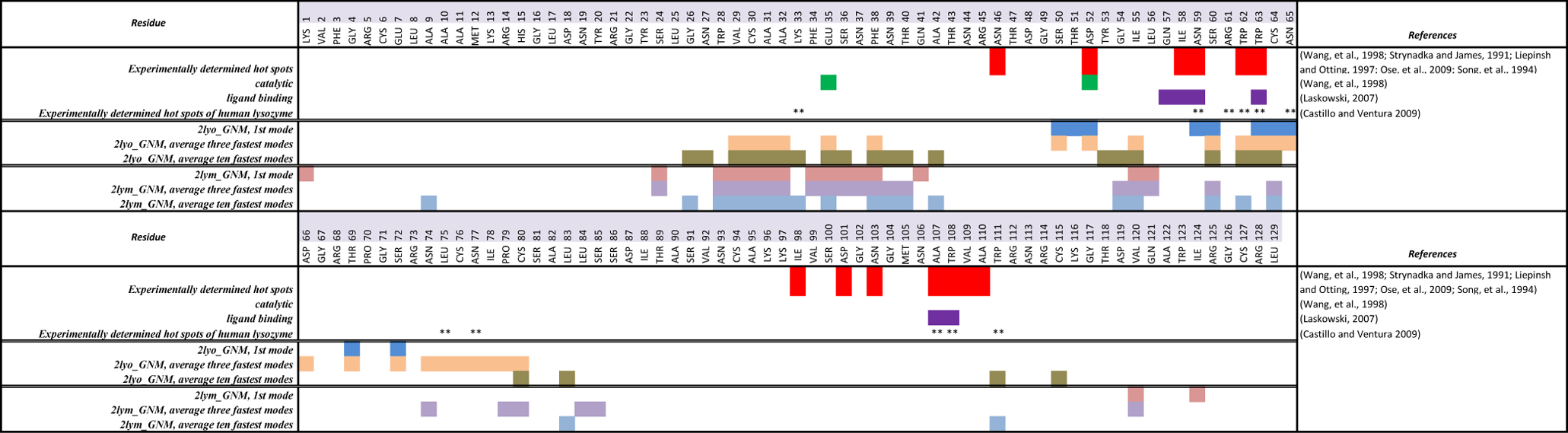

Supplement: Figure S11 — The GNM analysis performed on hen egg-white lysozyme (HEWL), a single polypeptide of 129 amino acids with the unbound (dark grey: 2 LYM [89] ) and bound (light grey: 2 LYO [83] ) structures. Experimentally determined hot spot residues [79], [85], [86], ligand binding sites and catalytic residues [83] are shown. The GNM suggested sites of the fastest mode and the average three fastest modes for2LYM and2LYO are marked. (TIF) [file pone.0074320.s011.tif]

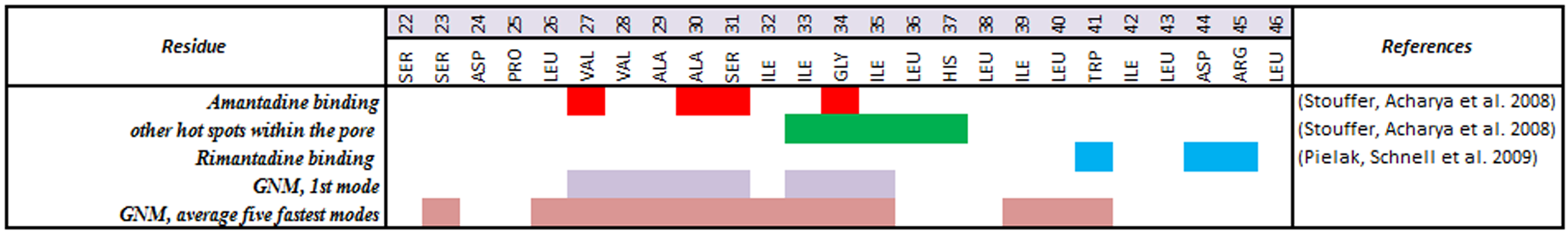

Supplement: Figure S12 — The GNM analysis performed on Influenza virus M2 proton channel, 3 BKD [95] . 3 BKD [95] is the drug unbound structure with four chains of 26 residues (22–47) each. 2KQT [105] is the solid state NMR structure of the amantadine bound M2 protein with four chains of 25 residues (22–46) each. 2RLF [91] is the rimantadine bound M2 protein structure with four chains that have 38 (23–60) residues each. Amantadine [95] and rimantadine [96] binding sites, and allosteric sites [98] are shown. The GNM suggested sites of the fastest mode and the average five fastest modes are marked. (TIF) [file pone.0074320.s012.tif]
