# Supplementary material for: Hot Spots in a Network of Functional Sites
Source: PLoS One. 2013 Sep 2;8(9):e74320. doi: 10.1371/journal.pone.0074320 (PMC3759471; doi:10.1371/journal.pone.0074320)
Supplement: File S1 — Supplementary Data. (DOCX) [file pone.0074320.s013.docx]

**SUPPORTING INFORMATION**

**Supplementary Data**

**Gaussian Network Model (GNM)**

The GNM [[53](#_ENREF_53),[54](#_ENREF_54)] is a simple elastic network where the proteins are represented by the nodes corresponding to alpha carbons and assumed to be connected by harmonic springs if within a cut-off radius (r_cut_). The potential function for a protein structure of N residues in the elastic network description is given as

$V_{GNM}=\frac{\gamma}{2}\left[\sum_{i,j}^{N} \mathbf{\Gamma}_{ij}\left[\begin{matrix} \left(\Delta X_{i}-\Delta X_{j}\right)^{2}+\left(\Delta Y_{i}-\Delta Y_{j}\right)^{2} \\ +\left(\Delta Z_{i}-\Delta Z_{j}\right)^{2} \end{matrix}\right] \right]$ (1)

Where γ is the spring constant and **Γ** is the Kirchhoff matrix, which is defined as

$\mathbf{\Gamma}_{ij}=\left\{\begin{matrix} \begin{matrix} -1 & \text{if }i\neq j\text{ and }R_{ij}\leq r_{c} \\ 0 & \text{if }i\neq j\text{ and }R_{ij}>r_{c} \end{matrix} \\ \begin{matrix} -\sum_{j,j\neq i}^{N} \mathbf{\Gamma}_{ij} & \text{if }i=j \end{matrix} \end{matrix}\right.$ (2)

R_ij_ is the distance between alpha carbon atoms i and j.

The mean square distance fluctuations of residues i and j, <Δ**R**_ij_^2^>, are calculated using equations 1 and 2 given in the main text. The summation in equation 2 goes over N-1 GNM modes for a protein of N residues. For the calculation of the fluctuations in the fast modes of motion, the eigenmodes with the highest eigenvalues are included in the summation and the corresponding <∆**R**_ij_^2^> are obtained. There are N^2^ mean-square distance fluctuation values. We sort these values in decreasing order, and find a threshold value T below which the correlation values stay constant. We increase T by 1%, and mark the residues i which have <∆**R**^2^_ij_> values greater than T*1.01. These residues are the suggested by the GNM and correspond to 11.4-16.9 % and 3.4-6.0% of total number of residues on the average for the unbound and bound cases in the dataset of this study, respectively.

**A case study**

1fkb with 107 residues have 13 hot spot residues determined experimentally [[32](#_ENREF_32)]. Figure S1 displays <∆**R**^2^_ij_> values for each residue i (with all j values) by GNM with the experimental hot spot residues (red dots). We sort all <∆**R**^2^_ij_> values in decreasing order. The point at which the <∆**R**^2^_ij_> value stays constant is taken as the threshold value “T”. “T” is increased by 1%, which is also demonstrated on the figure as a red line. The residues that have higher correlation value with any other residue in the structure than this threshold value are determined as a potential hot spot residue.

Table S4 displays the predicted residues by GNM and the experimental hot spot residues. Based on the fastest mode of motion, GNM suggests 16 residues as potential hot spot residues among which 4 overlap in exact match (bold) with the experimental hot spot residues. Up to two neighboring residues, 11 of 16 predicted hot spot residues (gray highlight) overlap 10 out of 13 experimental hot spot residues (gray highlight). Similarly, GNM suggests 21 residues as functionally plausible in the average three fastest modes, 5 overlaps in exact match (bold) and 13 up to two neighboring residues (gray) overlaps 11 out of 13 (stared) experimental hot spot residues. As can be observed, the false positives are highly around the hot spot residues.

**Statistical Analysis**

The statistical significance of the predicted residues is evaluated using Z-score analysis. Closeness centrality of residue k is defined as:

Where $C_{K}$is the distance between residue *k*, the predicted residue, and closest hot spot*,* $\bar{C}$ is the average distance to the closest hot spot over all protein residues and $\sigma$ is the corresponding standard deviation.

**Legends to Supplementary Tables**

**Table S1**. The summary of available servers and databases

| **Hot spot databases and prediction servers** | | **Input DATA** | **Method** | **Availability** | **Technique** |
| --- | --- | --- | --- | --- | --- |
| ASEdb (Bogan and Thorn, 1998) | http://nic.ucsf.edu/asedb/ | PDB ID | Experimental | Database | Alanine scanning |
| BID (Fischer, et al., 2003) | http://tsailab.tamu.edu/BID/ | PDB ID | Experimental | Database | Collection of several experimental techniques. |
| HotSprint (Guney, et al., 2008) | http://prism.ccbb.ku.edu.tr/hotsprint/ | PDB ID | Conservation, accessibility, residue propensity | Database | Empirical formula |
| FoldX (Guerois, et al., 2002) | http://foldx.crg.es/ | Complex structure | Energy-based | Tool and server | Computational alanine scanning |
| ISIS (Ofran and Rost, 2007) | http://cubic.bioc.columbia.edu/services/isis | Sequence | Sequence-based | Tool | Machine learning |
| K-FADE/ K-CON/ ROBETTA (KFC) (Darnell, et al., 2007) | http://kfc.mitchell-lab.org | Complex structure | Atomic contacts, residue size, H-bond | Server | Machine learning |
| Robetta (Kortemme and Baker, 2002) | <http://robetta.bakerlab.org/> | Complex structure | Energy-based | Server | Computational alanine scanning |
| MAPPIS (Shulman-Peleg, et al., 2007) | http://bioinfo3d.cs.tau.ac.il/MAPPIS | Complex structure | Evolutionary conservation | Server | Multiple alignments, 3D clustering |
| HotPoint (Tuncbag, et al., 2010) | http://prism.ccbb.ku.edu.tr/hotpoint | Complex structure | Empirical model | Server | Accessibility, knowledge-based potentials |
| pyDockNIP (Grosdidier and Fernandez-Recio, 2008) | http://mmb.pcb.ub.es/PyDock | Unbound protein structure | Energy-based | Server | Docking simulations |

**Table S2**. Datasets: (ASEdb[[62](#_ENREF_62)]; Kortemme and Baker [[33](#_ENREF_33)]; Guerois et al. [[32](#_ENREF_32)] and BID [[31](#_ENREF_31)]).

| **Protein ID** | **Chain ID** | **Function** | **Reference dataset** | **Number of hot spot residues** | **Hot spot residues** | **Total number of residues** |
| --- | --- | --- | --- | --- | --- | --- |
| 1amx | A | Bacterial adhesin | BID | 2 | 232ASN, 233TYR | 150 |
| 1bpi | A | Proteinase inhibitor (trypsin) | Guerois et al. | 5 | 4PHE,  19ILE, 24ASN, 44ASN, | 58 |
| 1bvc | A | Oxygen storage | Guerois et al. | 1 | 131MET | 153 |
| 1csp | A | Transcription regulation | Guerois et al. | 1 | 15PHE | 67 |
| 1fkb | A | isomerase | Guerois et al. | 13 | 2VAL,  4VAL, 23VAL, 24VAL, 50LEU, 60GLU, 63VAL, 75THR, 76ILE, 97LEU, 98VAL, 101VAL, 106LEU | 107 |
| 1fnh | A | Heparin and integrin binding | BID | 2 | 207ARG, 209ARG | 269 |
| 1iam | A | Viral protein receptor | BID | 1 | 34GLU | 185 |
| 1ifc | A | Lipid-binding protein | Guerois et al. | 1 | 64LEU | 262 |
| 1lrp | A | DNA binding regulatory protein | Guerois et al. | 1 | 40MET | 89 |
| 1nt3 | A | Hormone/growth factor/neuropeptide | ASEDB | 3 | 11TYR  68ARG  103ARG | 108 |
| 1pga | A | Immunoglobulin binding protein | Guerois et al. | 3 | 6ILE,  41GLY, 54VAL | 56 |
| 1rcb | A | Cytokine | ASEDB | 2 | 9GLU, 88ARG | 129 |
| 1rex | A | Hydrolase (o-glycosyl) | Guerois et al. | 3 | 23ILE,  56ILE,  89ILE | 130 |
| 1shg | A | Cytoskeleton | Guerois et al. | 6 | 23VAL, 25MET, 44VAL, 52PHE, 53VAL, 58VAL, | 57 |
| 1stn | A | Hydrolase(phosphoric diester) | Guerois et al. | 37 | 77ASP, 83ASP, 95ASP, 129GLU, 75GLU, 34PHE, 61PHE, 76PHE, 121HIS, 139ILE, 15ILE,  18ILE, 72ILE, 92ILE, 103LEU, 108LEU,  125LEU, 137LEU, 14LEU, 25LEU, 36LEU, 89LEU, 65MET, 98MET, 100ASN, 118ASN, 62THR,  104VAL, 111VAL, 23VAL, 39VAL, 66VAL, 74VAL, 99VAL, 27TYR, 54TYR, 91TYR | 136 |
| 1ycc | A | Electron transport (cytochrome) | Guerois et al. | 1 | 85LEU | 107 |
| 1ypc | I | Proteinase inhibitor(chymotrypsin) | Guerois et al. | 10 | 27LEU, 48ILE,  49ILE, 51LEU, 66VAL, 68LEU, 69PHE, 71ASP, 76ILE, 80PRO | 64 |
| 2lzm | A | Hydrolase (o-glycosyl) | Guerois et al. | 19 | 149VAL, 104PHE, 153PHE, 100ILE, 17ILE,  27ILE,  29ILE,  50ILE,  58ILE, 118LEU, 121LEU, 133LEU, 33LEU, 66LEU, 7LEU, 84LEU, 91LEU, 99LEU, 106MET | 164 |
| 3bct | A | Armadillo repeat | BID | 9 | 253PHE, 260HIS, 292LYS, 338TRP, 342ARG, 383TRP, 435LYS, 469ARG, 508LYS | 444 |
| 3il8 | A | Preliminary | ASEDB | 3 | 4GLU*,  5LEU,  6ARG | 68 |
| 4fgf | A | Growth factor | ASEDB | 4 | 24TYR, 96GLU, 103TYR, 140LEU | 124 |
| 4lyz | A | Hydrolase | Guerois et al. | 1 | 55ILE | 129 |
| 5ebx | A | Toxin | ASEDB | 2 | 10GLN, 47LYS | 62 |
| 1jic | A | DNA-binding protein/DNA | Guerois et al. | 5 | 3VAL, 14VAL, 29ILE, 31PHE, 54LEU | 62 |
| 1u9p | A | Endonuclease | ASEDB | 3 | 29TYR, 35ASP, 39ASP | 89 |
| 1imp | A | Immune system | ASEDB | 6 | 33LEU, 34VAL, 41GLU, 50SER, 51ASP, 55TYR | 86 |
| 1q1p | A | Cell adhesion protein | BID | 2 | 31GLU, 89GLU | 212 |
| 2igg | A | Complex (antibody/antigen) | Kortemme and Baker | 4 | 32GLU, 36LYS, 40ASN, 48TRP | 64 |
| 1h4u | A | Basement membrane | BID | 5 | 427ASP, 429HIS, 431TYR, 616GLU, 620ARG | 245 |
| 1k51 | A | Protein binding | Guerois et al. | 8 | 6ILE,  10LEU, 12PHE, 22PHE, 36TYR, 40LEU, 58LEU, 60ILE | 72 |
| 1ck1 | A | Complex (toxin/receptor) | ASEDB | 4 | 23ASN, 90TYR, 91VAL, 210GLN | 239 |
| 1mjn | A | Immune system | BID | 3 | 137ASP, 206THR, 239ASP | 179 |
| 1iyy | A | Hydrolase (endoribonuclease) | Guerois et al. | 4 | 76ASP, 44ASN, 81ASN, 39PRO | 104 |

*Missing in the PDB structure but reported in ASEdb[[62](#_ENREF_62)], not included in the calculations.

**Table S3**. Datasets of complex structures (ASEdb[[62](#_ENREF_62)]; Kortemme and Baker [[33](#_ENREF_33)]; Guerois et al. [[32](#_ENREF_32)] and BID[[31](#_ENREF_31)]). The complex structures are used as the hot spot data information source.

| **Protein ID** | **Chain ID** | **Function** | **Unbound Structure** | **Chain ID** | **Reference dataset** | **RMSD (Å)** | **Number of hot spot residues** | **Hot spot residues** |
| --- | --- | --- | --- | --- | --- | --- | --- | --- |
| 1brs | D | Endonuclease | 1ab7 | A | ASEDB | 1,35 | 3 | TYR29,  ASP35,  ASP39 |
| 1bxi | A | Immune system | 1imp | A | ASEDB | 1,43 | 6 | LEU33,  VAL34,  GLU41,  SER50,  ASP51,  TYR55 |
| 1edh | A | Cell adhesion protein | 1q1p | A | BID | 1,14 | 2 | GLU31, GLU89 |
| 1fcc | C | Complex (antibody/antigen) | 2igg | A | Kortemme and Baker | 1,57 | 4 | GLU27,  LYS31,  ASN35, TRP43 |
| 1gl4 | A | Basement membrane | 1h4u | A | BID | 0,67 | 5 | ASP427,  HIS429,  TRY431,  GLU616,  ARG620 |
| 1hz6 | A | Protein binding | 1k51 | A | Guerois et al. | 1,2 | 8 | ILE6,  LEU10,  PHE12,  PHE22,  TYR36,  LEU40,  LEU58,  ILE60 |
| 1jck | B | Complex (toxin/receptor) | 1ck1 | A | ASEDB | 0,8 | 4 | ASN23,  TRY90,  VAL91,  GLN210 |
| 1mq8 | B | Immune system | 1mjn | A | BID | 1,71 | 3 | ASP137,  THR206,  ASP239 |
| 1rn1 | A | Hydrolase  (endoribonuclease) | 1iyy | A | Guerois et al. | 1,88 | 4 | ASP76,  ASN44,  ASN81,  PRO39 |

**Table S4.** GNM results for the fastest and the average fastest three modes of motion and experimental hot spot residues [[32](#_ENREF_32)] for 1fkb.

| Fastest Mode | Average of fastest three modes | Hot Spot Residues |
| --- | --- | --- |
| **4** | **4** | 2* |
| 5 | 6 | 4* |
| 6 | 22 | 23* |
| 7 | **24** | 24* |
| **23** | 25 | 50* |
| 26 | 26 | 60* |
| 28 | 29 | 63* |
| 58 | 30 | 75* |
| 67 | 45 | 76* |
| 70 | 46 | 97 |
| 72 | 47 | 98 |
| 74 | **50** | 101* |
| **76** | 57 | 106* |
| 99 | 62 |  |
| **101** | 67 |  |
| 103 | 70 |  |
|  | **76** |  |
|  | 78 |  |
|  | **101** |  |
|  | 103 |  |
|  | 104 |  |

**Table S5**.The GNM performance values of the bound dataset

| **NO GNM** | | | | | **RSA** | | | | **CONSERVATION** | | | | **RSA & CONSERVATION** | | | | |
| --- | --- | --- | --- | --- | --- | --- | --- | --- | --- | --- | --- | --- | --- | --- | --- | --- | --- |
|  |  |  |  |  | 90 | 37 | 4 | 39 | 79 | 42 | 4 | 43 | 69 | | 55 | 5 | 56 |
| ***GNM modes*** | **EXACT** | | | | **EXACT & RSA** | | | | **EXACT & CONSERVATION** | | | | **EXACT & RSA & CONSERVATION** | | | | |
|  | ***S*** | ***C*** | ***P*** | ***A*** | ***S*** | ***C*** | ***P*** | ***A*** | ***S*** | ***C*** | ***P*** | ***A*** | ***S*** | ***C*** | | ***P*** | ***A*** |
| ***1*** | 15 | 89 | 4 | 87 | 13 | 91 | 4 | 88 | 10 | 95 | 6 | 92 | 8 | 95 | | 5 | 92 |
| ***2*** | 33 | 89 | 8 | 87 | 33 | 90 | 9 | 88 | 26 | 95 | 13 | 93 | 26 | 95 | | 13 | 93 |
| ***3*** | 36 | 89 | 9 | 87 | 31 | 89 | 8 | 87 | 21 | 94 | 10 | 92 | 21 | 95 | | 11 | 92 |
| ***1-3*** | 31 | 81 | 5 | 80 | 36 | 82 | 6 | 81 | 28 | 90 | 8 | 88 | 28 | 90 | | 8 | 89 |
| ***1-5*** | 38 | 83 | 6 | 81 | 38 | 82 | 6 | 81 | 28 | 90 | 8 | 88 | 28 | 90 | | 8 | 88 |
| **GNM modes** | **NEIGHBOR 1** | | | | **NEIGHBOR 1& RSA** | | | | **NEIGHBOR 1 & CONSERVATION** | | | | **NEIGHBOR 1 & RSA & CONSERVATION** | | | | |
|  | ***S*** | ***C*** | ***P*** | ***A*** | ***S*** | ***C*** | ***P*** | ***A*** | ***S*** | ***C*** | ***P*** | ***A*** | ***S*** | | ***C*** | ***P*** | ***A*** |
| ***1*** | 18 | 89 | 5 | 87 | 15 | 91 | 5 | 88 | 13 | 95 | 7 | 92 | 10 | | 95 | 6 | 93 |
| ***2*** | 36 | 89 | 9 | 87 | 36 | 90 | 10 | 89 | 28 | 95 | 14 | 93 | 28 | | 95 | 15 | 93 |
| ***3*** | 36 | 89 | 9 | 87 | 36 | 89 | 9 | 88 | 26 | 94 | 12 | 92 | 26 | | 95 | 13 | 93 |
| ***1-3*** | 41 | 82 | 6 | 80 | 41 | 83 | 7 | 81 | 33 | 90 | 10 | 89 | 33 | | 91 | 10 | 89 |
| ***1-5*** | 46 | 83 | 8 | 82 | 46 | 83 | 8 | 81 | 36 | 90 | 10 | 89 | 36 | | 90 | 10 | 89 |
| **GNM modes** | **NEIGHBOR 2** | | | | **NEIGHBOR 2& RSA** | | | | **NEIGHBOR 2 & CONSERVATION** | | | | **NEIGHBOR 2 & RSA & CONSERVATION** | | | | |
|  | ***S*** | ***C*** | ***P*** | ***A*** | ***S*** | ***C*** | ***P*** | ***A*** | ***S*** | ***C*** | ***P*** | ***A*** | ***S*** | | ***C*** | ***P*** | ***A*** |
| ***1*** | 21 | 89 | 5 | 87 | 18 | 91 | 6 | 89 | 13 | 95 | 7 | 92 | 10 | | 95 | 6 | 93 |
| ***2*** | 46 | 89 | 12 | 88 | 46 | 90 | 13 | 89 | 31 | 95 | 15 | 93 | 31 | | 95 | 16 | 93 |
| ***3*** | 38 | 89 | 10 | 88 | 38 | 89 | 10 | 88 | 28 | 94 | 14 | 92 | 28 | | 95 | 15 | 93 |
| ***1-3*** | 49 | 82 | 8 | 81 | 49 | 83 | 8 | 82 | 36 | 90 | 10 | 89 | 36 | | 91 | 11 | 89 |
| ***1-5*** | 51 | 83 | 9 | 82 | 51 | 83 | 8 | 82 | 38 | 90 | 11 | 89 | 38 | | 90 | 11 | 89 |

**Table S6**.The GNM performance values of the complex dataset

| **NO GNM** | | | | | **RSA** | | | | **CONSERVATION** | | | | **RSA & CONSERVATION** | | | | |
| --- | --- | --- | --- | --- | --- | --- | --- | --- | --- | --- | --- | --- | --- | --- | --- | --- | --- |
|  |  |  |  |  | 90 | 37 | 4 | 39 | 79 | 42 | 4 | 43 | 69 | | 55 | 5 | 56 |
| ***GNM modes*** | **EXACT** | | | | **EXACT & RSA** | | | | **EXACT & CONSERVATION** | | | | **EXACT & RSA & CONSERVATION** | | | | |
|  | ***S*** | ***C*** | ***P*** | ***A*** | ***S*** | ***C*** | ***P*** | ***A*** | ***S*** | ***C*** | ***P*** | ***A*** | ***S*** | ***C*** | | ***P*** | ***A*** |
| ***1*** | 15 | 97 | 16 | 95 | 13 | 98 | 15 | 95 | 10 | 98 | 17 | 96 | 8 | 98 | | 14 | 96 |
| ***2*** | 33 | 98 | 36 | 96 | 33 | 98 | 39 | 96 | 26 | 99 | 38 | 97 | 26 | 99 | | 40 | 97 |
| ***3*** | 36 | 98 | 33 | 96 | 31 | 98 | 29 | 96 | 21 | 98 | 30 | 96 | 21 | 99 | | 32 | 96 |
| ***1-3*** | 31 | 96 | 18 | 94 | 36 | 96 | 22 | 94 | 28 | 97 | 24 | 95 | 28 | 97 | | 25 | 95 |
| ***1-5*** | 38 | 96 | 23 | 94 | 38 | 96 | 23 | 94 | 28 | 97 | 24 | 95 | 28 | 97 | | 24 | 95 |
| **GNM modes** | **NEIGHBOR 1** | | | | **NEIGHBOR 1& RSA** | | | | **NEIGHBOR 1 & CONSERVATION** | | | | **NEIGHBOR 1 & RSA & CONSERVATION** | | | | |
|  | ***S*** | ***C*** | ***P*** | ***A*** | ***S*** | ***C*** | ***P*** | ***A*** | ***S*** | ***C*** | ***P*** | ***A*** | ***S*** | | ***C*** | ***P*** | ***A*** |
| ***1*** | 18 | 98 | 18 | 95 | 15 | 98 | 18 | 95 | 13 | 99 | 22 | 96 | 10 | | 99 | 18 | 96 |
| ***2*** | 36 | 98 | 39 | 96 | 36 | 98 | 42 | 97 | 28 | 99 | 42 | 97 | 28 | | 99 | 44 | 97 |
| ***3*** | 36 | 98 | 33 | 96 | 36 | 98 | 34 | 96 | 26 | 99 | 37 | 96 | 26 | | 99 | 40 | 97 |
| ***1-3*** | 41 | 96 | 24 | 94 | 41 | 96 | 25 | 95 | 33 | 97 | 29 | 96 | 33 | | 98 | 30 | 96 |
| ***1-5*** | 46 | 96 | 27 | 95 | 46 | 96 | 28 | 95 | 36 | 97 | 30 | 96 | 36 | | 97 | 30 | 96 |
| **GNM modes** | **NEIGHBOR 2** | | | | **NEIGHBOR 2& RSA** | | | | **NEIGHBOR 2 & CONSERVATION** | | | | **NEIGHBOR 2 & RSA & CONSERVATION** | | | | |
|  | ***S*** | ***C*** | ***P*** | ***A*** | ***S*** | ***C*** | ***P*** | ***A*** | ***S*** | ***C*** | ***P*** | ***A*** | ***S*** | | ***C*** | ***P*** | ***A*** |
| ***1*** | 21 | 98 | 21 | 95 | 18 | 98 | 21 | 95 | 13 | 99 | 22 | 96 | 10 | | 99 | 18 | 96 |
| ***2*** | 46 | 99 | 50 | 97 | 46 | 99 | 55 | 97 | 31 | 99 | 46 | 97 | 31 | | 99 | 48 | 97 |
| ***3*** | 38 | 98 | 35 | 96 | 38 | 98 | 37 | 96 | 28 | 99 | 41 | 97 | 28 | | 99 | 44 | 97 |
| ***1-3*** | 49 | 96 | 28 | 95 | 49 | 96 | 30 | 95 | 36 | 98 | 31 | 96 | 36 | | 98 | 32 | 96 |
| ***1-5*** | 51 | 96 | 30 | 95 | 51 | 96 | 31 | 95 | 38 | 98 | 33 | 96 | 38 | | 98 | 33 | 96 |

- Labels S, C, P and A refer to sensitivity, specificity, precision and accuracy respectively. GNM modes 1-3 and 1-5 refer to the average of the three and the five fastest modes respectively. The reported values are percentages.
